# Supplementary material for: Mitochondrial Fission Governed by Drp1 Regulates Exogenous Fatty Acid Usage and Storage in Hela Cells
Source: Metabolites. 2021 May 18;11(5):322. doi: 10.3390/metabo11050322 (PMC8157282; doi:10.3390/metabo11050322)
Supplement: Supplementary file 1 [file metabolites-11-00322-s001.zip › Supplemental Figure legends.pdf]

Supplemental Figure 1. Palmitate changes mitochondrial morphology. The areas (A), perimeters (B) and aspect ratios (major axis/minor axis) (C) of individual mitochondria in cells incubated in BM, BM+PA, BM+OA for 4 hours. (D) The number of mitochondria in a cell when incubated in BM, BM+PA and BM+OA for 4 hours. n=44-48; 3 independent experiements. Data are expressed as mean ± S.E.M. Ordinary one-way ANOVA-Tukey's multiple comparisons test.

Supplemental Figure 2. Palmitate affects mitochondrial respiration. (A) Representative images of live HeLa cells incubated in 5mM glucose (Glc; BM-Gln) and 5mM glucose+100μM palmitate (Glc+PA) for 4 hours after 2-hour incubation in Live Cell Imaging Solution. Mitochondria are labeled with MitoTracker Orange. Scale bars=10μm. (B) Quantifications of mitochondrial morphology of cells incubated in different nutrients. n=51-55. 3 experiments. Seahorse experiment was performed to measure basal respiration (C), ATP-linked respiration (D), non-mitochondrial oxygen consumption (E) after cells were incubated in Glc, Glc+PA, BM, BM+PA for 4 hours. (F) Measurements of the contribution of fatty acids to TCA cycle using ^{13}C-glucose after 4-hour incubation. (G) Measurements of mitochondrial mass using MitoTracker Green after 4-hour incubation. Normalized to cells incubated in the growth media (CTRL). (B-G) Data are expressed as mean ± S.E.M. Ordinary one-way ANOVA-Tukey's multiple comparisons test.

Supplemental Figure 3. The effect of DRP1 KD on mitochondria and lipid droplets**.** (A) Western blot of DRP1. Cells were transfected with 40nM of NC siRNA or DRP1 siRNA. (B) Representative images of cells transfected with NC siRNA or DRP1 siRNA after 4-hour incubation in BM. No differences in mitochondrial morphology or the amount of lipid droplets were observed between cells transfected with NC siRNA and DRP1 siRNA. Mitochondria are labeled with MitoTracker DeepRed and lipid droplets are labeled with LipidTOX Green. Scale bars=10μm. (C) Measurements of mitochondrial mass using MitoTracker Green in cells transfected with NC siRNA or DRP1 siRNA after 4-hour incubation in BM or BM+PA. Normalized to the control cells incubated in BM. Data are expressed as mean ± S.E.M. 2way ANOVA-Sidak's multiple comparison test. **p<0.01.

Supplemental Figure 4. The effect of DRP1 KD on cellular metabolism. (A) Non-mitochondrial oxygen consumption rate after 1-hour incubation (n=11-13). Data are expressed as mean ± S.E.M. 2way ANOVA-Sidak's multiple comparison test. (B) RT-qPCRs were performed after 1-hour incubation in BM or BM+PA to measure expression levels of following genes in cells transfected with NC siRNA or DRP1 siRNA: 3-ketoacyl-CoA thiolase (*ACAA1*), fatty acyl-CoA oxidase (*ACOX1*), diacylglycerol acyltransferase 1 (*DGAT1*) peroxisomal bifunctional enzyme (*EHHADH*), tRNA-Leu (for mtDNA). n=5. Data are expressed as mean ± S.E.M. 2way ANOVA-Sidak's multiple comparison test. (C) Representative EM image of ER-mitochondrion contact. Control and DRP1 KD cells were incubated with BM or BM+PA for 1 hour before fixation. ER is shaded with pink and mitochondrion is shaded with blue. The number of ER-mitochondrion contact was divided by cytoplasm area (μm^2). n=7-11. Data are expressed as mean ± S.E.M. 2way ANOVA-Sidak's multiple comparison test. Scale bar=1μm.

Supplemental Figure 5. CPT1A OE does not alter the fatty acid storage/usage balance. (A) Western blot of CPT1A. After the transfection with CPT1A plasmid and singe-cell sorting, the colony with the highest expression of CPT1A (indicated by the arrow) was selected. Individual lanes were whole cell lysate of individual colonies. (B) Quantifications of the area of lipid droplets per cell in WT HeLa (CTRL; n=42) and CPT1A overexpressing HeLa cells (CPT1A OE; n=42) after 4-hour incubation in BM+PA. 3 independent experiments. Data are expressed as mean ± S.E.M. Unpaired two-tailed t-test. (C) Measurements of contribution of fatty acid to TCA cycle using ^{13}C-glucose after 4-hour incubation in CTRL and CPT1A OE cells. Data are expressed as mean ± S.E.M. 2way ANOVA-Sidak's multiple comparison test. (D) ATP-linked respiration of WT HeLa cells (CTRL) and CPT1A OE cells in BM and BM+PA measured with

Seahorse. Data are expressed as mean ± S.E.M. 2way ANOVA-Sidak's multiple comparison test. (E) The xy-plot of C12-Mito % and C12-LD % in WT HeLa cells and CPT1A OE cells. No populational shift was observed in both incubation times. The difference in the slopes between CTRL and CPT1A OE observed in 1-hour incubation disappears in 4-hour incubation. (F) The xy-plot of C12-Mito % and C12-LD % in cells transfected with NC siRNA and cells transfected with DRP1 siRNA. The separation of population remained the same and the difference in the slope was still observed regardless of incubation time.

Supplemental Figure 6. Morphological changes in mitochondria in CPT1A knockdown cells. (A) CPT1A silencing confirmed by RT-qPCR. Cells were transfected with 40nM of non-coding (NC) siRNA or CPT1A siRNA. n=3 Data are expressed as mean ± S.E.M. 2way ANOVA-Sidak's multiple comparison test. ****p<0.0001, ***p<0.001 (B) Quantification of mitochondrial morphology. NC siRNA: BM (n=46), BM+PA (n=46); CPT1A siRNA: BM (n=45), BM+PA (n=47). 3 independent experiments. Data are expressed as mean ± S.E.M. 2way ANOVA-Sidak's multiple comparison test. ****p<0.0001, **p<0.01 (BM vs BM+PA); ##p<0.01 # p<0.05 (CTRL vs CPT1A OE). (C) Quantification of the area of lipid droplets per cells transfected with NC siRNA (n=46) and CPT1A siRNA (n=47) after 4-hour incubation in BM+PA. 3 independent experiments. Data are expressed as mean ± S.E.M. Unpaired two-tailed t-test.
