# Supplementary figures and images for: Mitochondrial Fission Governed by Drp1 Regulates Exogenous Fatty Acid Usage and Storage in Hela Cells
Source: Metabolites. 2021 May 18;11(5):322. doi: 10.3390/metabo11050322 (PMC8157282; doi:10.3390/metabo11050322)

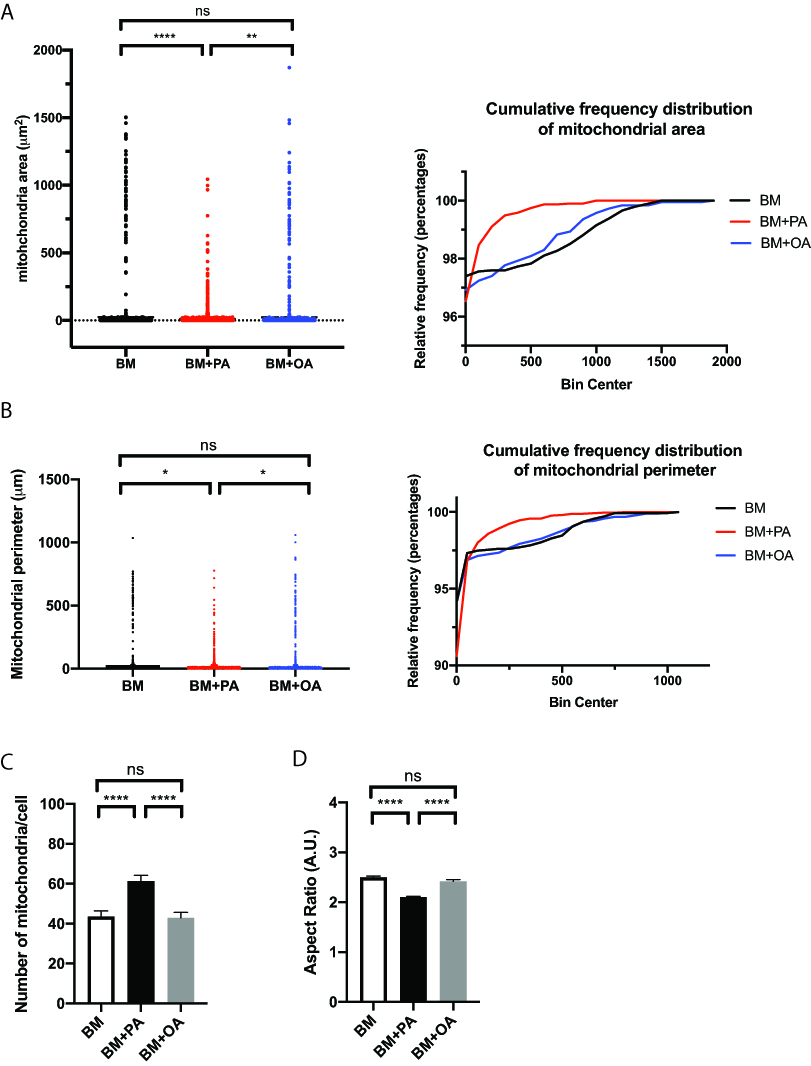

Supplement: Supplementary file 1 [file metabolites-11-00322-s001.zip › FigS1.tif]

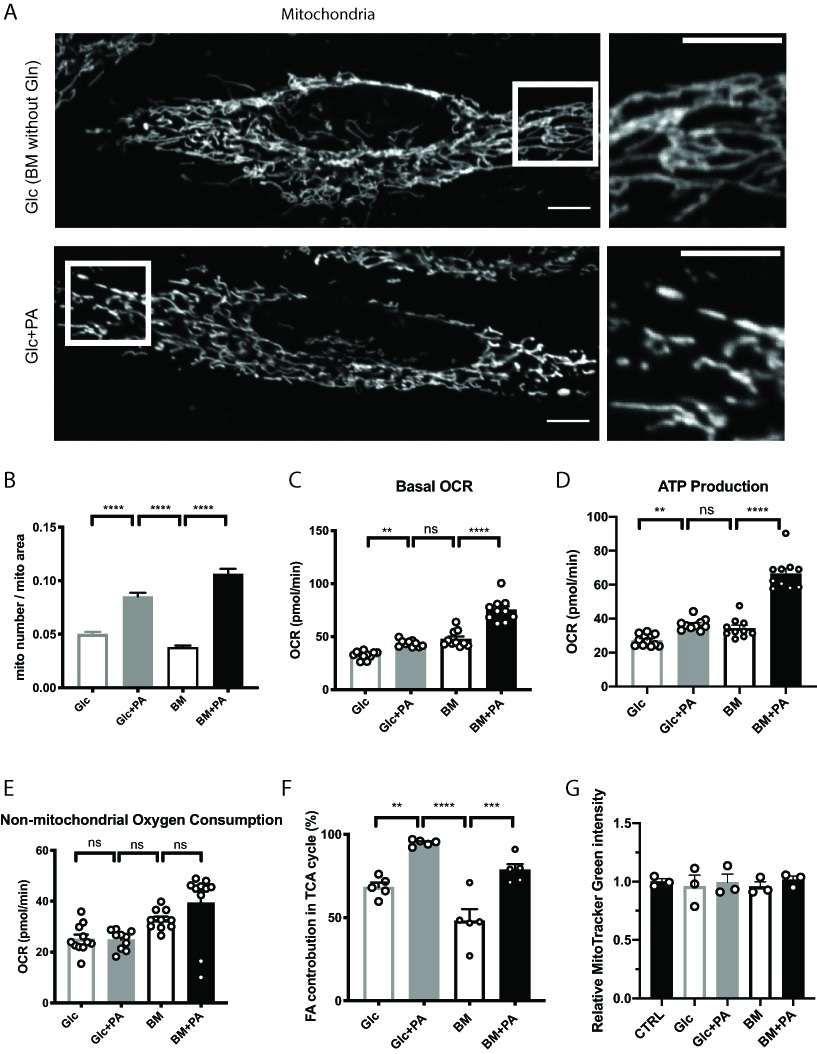

Supplement: Supplementary file 1 [file metabolites-11-00322-s001.zip › FigS2.tif]

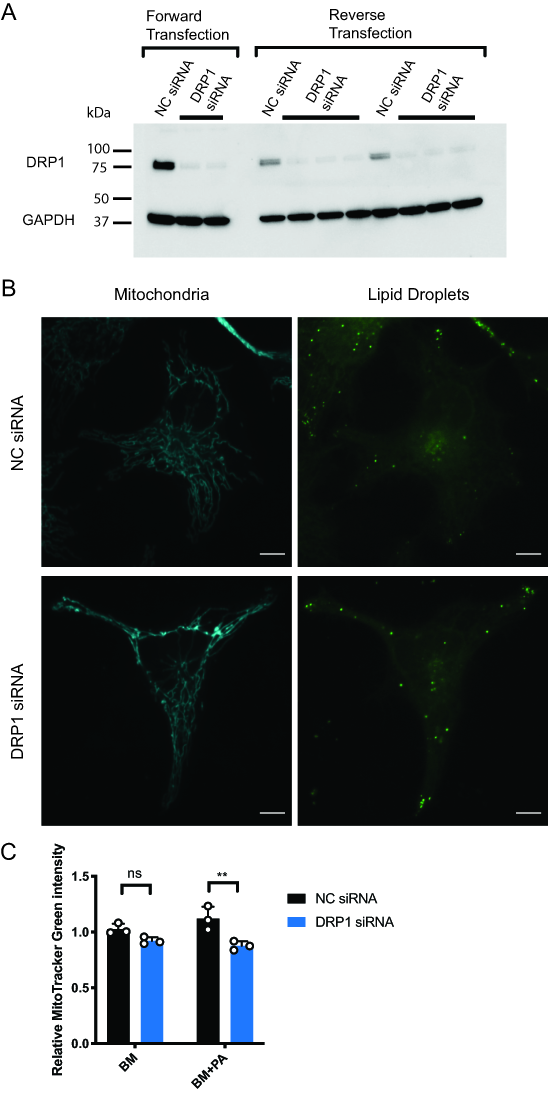

Supplement: Supplementary file 1 [file metabolites-11-00322-s001.zip › FigS3.tif]

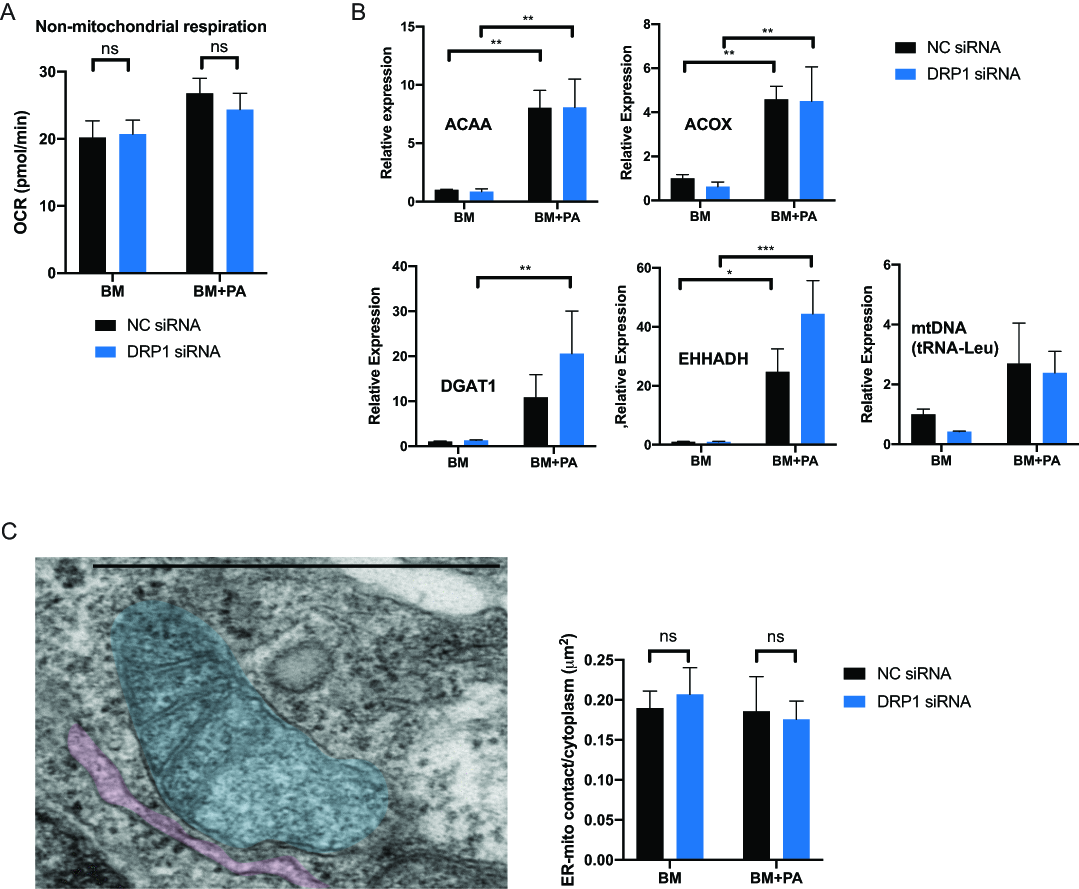

Supplement: Supplementary file 1 [file metabolites-11-00322-s001.zip › FigS4.tif]

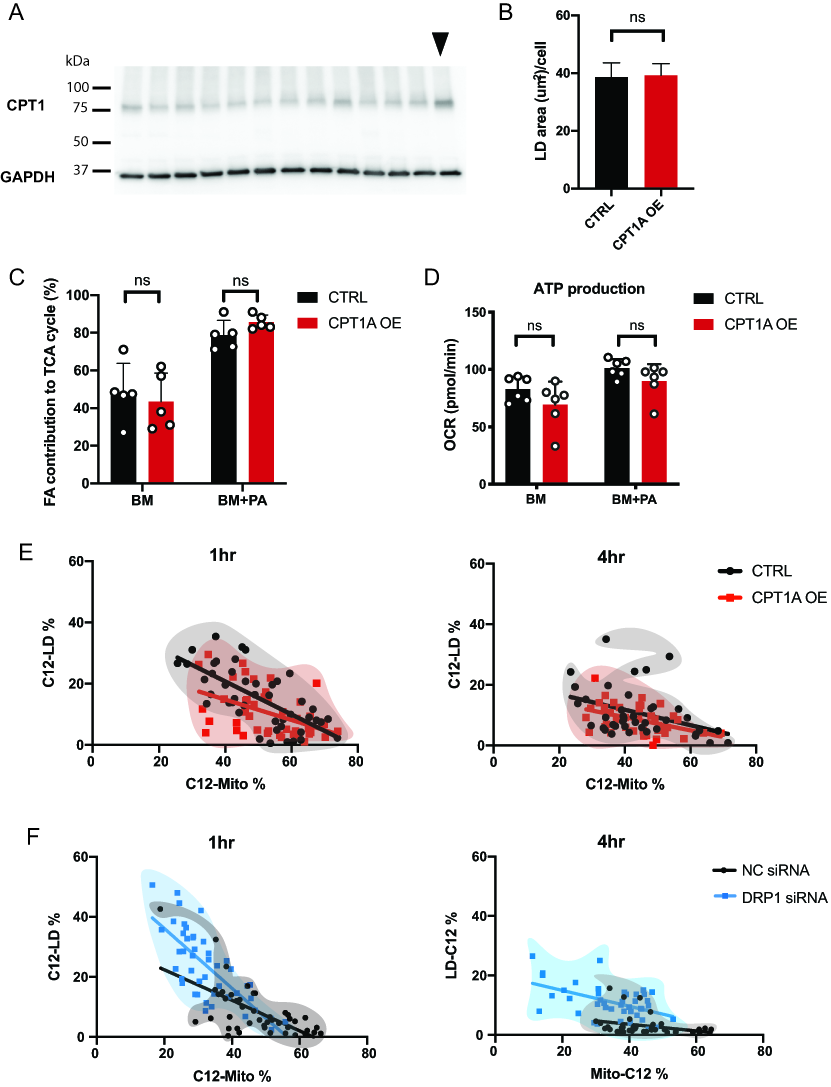

Supplement: Supplementary file 1 [file metabolites-11-00322-s001.zip › FigS5.tif]

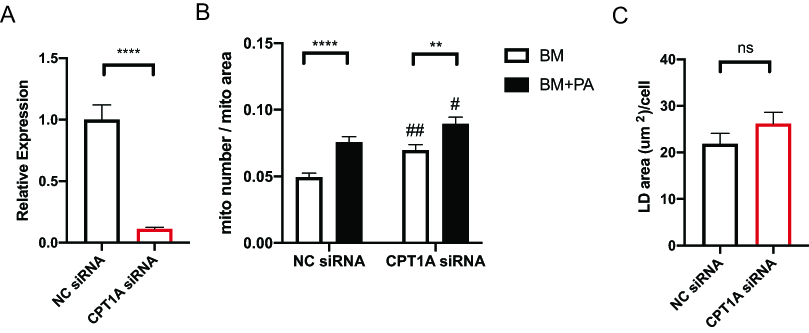

Supplement: Supplementary file 1 [file metabolites-11-00322-s001.zip › FigS6.tif]
